# Emotional State Measurement Trial (EMOPROEXE): A Protocol for Promoting Exercise in Adults and Children with Cerebral Palsy

**DOI:** 10.3390/jpm14050521

**Published:** 2024-05-14

**Authors:** Isabel M. Gómez-González, Juan A. Castro-García, Manuel Merino-Monge, Gemma Sánchez-Antón, Foad Hamidi, Alejandro Mendoza-Sagrera, Alberto J. Molina-Cantero

**Affiliations:** 1 Departamento de Tecnología Electrónica, E.T.S.I. Informática, Universidad de Sevilla, 41012 Sevilla, Spain; jacastro@us.es (J.A.C.-G.); manmermon@dte.us.es (M.M.-M.); gemma@us.es (G.S.-A.); almolina@us.es (A.J.M.-C.); 2 Instituto Universitario de Investigación de Ingeniería Informática, Universidad de Sevilla, 41012 Sevilla, Spain; 3 Information Systems Department, University of Maryland, College Park, MD 20742, USA; foadhamidi@umbc.edu; 4 Asociación Sevillana de Parálisis Cerebral, 41704 Dos Hermanas, Spain; aspacesevilla@aspace.org

**Keywords:** physiological computing, affective computing, sport promotion

## Abstract

Background: The protocol described in this paper is part of a research project coordinated between three Spanish universities, where a technology aimed at improving the quality of life of people with cerebral palsy will be developed. Part of the proposed technology will consist of an interface and a series of applications to increase motivation for daily physical activity. The basis of these developments is the measurement of the emotional state of the subjects. Methods: The experimental protocol is designed with two research objectives, on the one hand to identify the emotional state through physiological signals, and on the other to determine whether music can be a motivating factor to promote physical activity. It is specifically designed for subjects with cerebral palsy, taking into account the special characteristics of this population. These are people with whom it is difficult to use questionnaires to have a basis to contrast with the measured physiological signals, so measurements must be taken in carefully chosen daily-life situations. Discussion: We hope our findings show which physiological parameters are the most robust to measure the emotional state and how to design rehabilitation and physical activity promotion routines that are motivating, in addition to being able to avoid risk factors during the performance of these routines. Trial registration: NCT05621057.

## 1. Introduction

Physical activity is recommended at all ages and for both healthy people and people with functional diversity. The World Health Organization recommends it and even places special emphasis on children and adolescents. In the particular case of people with CP, it is of particular importance. It has demonstrated benefits for motor functions, including enhancing gait speed [1], spasticity [2], balance [3] and muscle strength [4]. It is therefore desirable to find procedures that increase motivation and help to improve the times and routines that this population dedicates to exercise. Detecting the emotional state and fatigue that a subject may have is important, although in this type of population the detection of these states is difficult given individuals’ communication difficulties.

The objective of this experimental protocol is to determine the relationship between physiological measurements and emotional state in children and adults with cerebral palsy (CP). Our research goal is to collect objective and robust information (based on biosignals) about the user’s condition to improve the effectiveness of rehabilitation therapies and the promotion of physical activity, and to determine whether music can improve these outcomes. The knowledge of the user’s emotional state is important to conduct the appropriate activities for their rehabilitation.

The experimentation will be conducted with a group of users belonging to the Association of People with Cerebral Palsy of Seville (ASPACE) and the Director Mercedes Sanroma Special Education School of Seville.

The methodology will be applied to groups of users of different ages with different capacities. For this reason, we have designed a flexible methodology that proposes different tasks according to the context. Certain tasks require physical effort, a factor that must be considered given the potential disruption that it may cause in the data collected [5].

Having an emotional characterization of the user based on their physiological measurements will enable us to obtain a real-time evaluation of the user’s emotions while tasks are being performed. Consequently, activity can be improved by adjusting the required parameters for effective computation, leading to better results.

The challenge we propose to address is that people with severe disabilities struggle to disclose and identify emotional states, which eliminates the possibility of utilizing self-assessment tests for comparison with the collected measures. This requires us to rely on their caregivers, or, alternatively/additionally, to implement measures in everyday scenarios or contexts where the emotional state elicited in the individual is understood.

This experimentation is part of a subproject called AAI (Augmentative Affective Interface), which in turn is part of the AIR4DP (Artificial Intelligence and Robotic Assistive Technology devices for Disabled People) coordinated project that is funded by the Ministry of Science, Innovation and Universities (I+D+i Projects 2019— Society Challenges). The main expected result of the AIR4DP project is the implementation of assistive technology that allows incorporating the latest advances in artificial intelligence (AI) to improve the quality of life of people with disabilities. The AAI subproject seeks an improvement in users’ interaction with systems achieved through knowledge of their emotional state based on physiological measurements and imagery. If this knowledge were injected into a robotic platform or a software platform used in rehabilitation therapies, a greater immersion and motivation of the subject in the proposed activities could be achieved.

The structure of the current paper is as follows. First, a review of the state of the art is presented, taking into account current knowledge on the factors involved in the study of emotional state, the target population (people with CP) and the influence of music on the state of mind. Subsequently, in Section 3, the methodology of the proposed experimentation is described. The type of study and the criteria for the inclusion and exclusion of participants are indicated. The instruments used to carry out the measurements to determine the state of the subjects are listed and explained; these instruments include measurement scales and physiological measurements. Section 4 shows that the chosen sample size is adequate and explains the statistical analysis that will be carried out with the collected data. Finally, in Section 5, we conclude by establishing the importance of designing an experimental protocol in accordance with the characteristics of the subjects that allows for the objectives set out in the protocol to be met.

## 2. Review of the State of the Art

In order to review existing relevant research and create a knowledge base to inform our study, we conducted three searches of research (SCOPUS and IEEE Xplore) with the following keywords:

1. Emotion AND detection AND physical AND activity. We used these keywords to explore methodologies that allow the detection of emotional state during physical activities, or the proposal of physical activity according to a specific emotional state.
2. Emotion AND detection AND cerebral AND palsy. With this search, we intended to detect studies related to the determination of emotional state in people with cerebral palsy. The special characteristics of this population mean that the usual methodologies are not fully applicable, so it is of interest to study cases where this type of measurement was made.
3. Emotion AND elicitation AND music. Music provokes emotions in subjects. The qualities of sound, such as frequency, timbre, duration and intensity, influence the induced emotions, hence its use in therapies. Music can be a way of bringing a subject to a desired emotional state to correlate parameters measured in said state, or music can be used as a form of motivation to carry out activities.

In [6], a health assistant system for subjects with depression was proposed. The system has three facets: the first remembers the taking of medications; the second measures the emotional state of the subjects, focusing mainly on electroencephalography (EEG); and the third proposes exercises based on the previous determination of the emotional state. In [7], physiological signals, specifically electrodermal activity (EDA), also called galvanic skin response (GSR), were used to detect the state of relaxation in subjects. In [8], an experiment with 16 participants was carried out to measure stress in real life. A smartwatch measured temperature, pulse, GSR and acceleration data. Contextual weather information was also measured. The incorporation of the environment measurement increased the precision in the detection of the stress state. In addition, these measures were based on responses to stress self-assessment questionnaires. In [9], a system was proposed to motivate and monitor the physical activity of older adults, based on the emotional state for activity proposals. The system relied on a database called AMIGOS [10]. We present the questionnaires used for both users and caregivers to assess their experience using the system: the questionnaire for users is shown in Table 1 and that for caregivers in Table 2.

In [11], a robotic platform was proposed to encourage physical activity in older adults, with their emotional states measured through images. In [12], cognitive fatigue was measured using a mobile application and 18 people participated in the experiment. The measure is based on the responses of the subjects to mobile games, a questionnaire and software-based facial recognition. In [13], different methods for the classification and processing of the EEG signal were evaluated to detect emotional states caused by a game. In [14], to motivate older people to exercise using a walker, a three-dimensional model of human fitness (arousal, pleasure and fatigue) was proposed. This measurement was used for the control of the walker. Wearable devices were used to measure physiological parameters (EEG, ECG, EMG). The results showed that the proposed solution increased the level of motivation to exercise and prevented muscle damage. In [15], a metric called cross-sample entropy was proposed to analyse EEG signal recordings. The results showed that the extracted parameters were useful to distinguish the states of calmness and distress in the subjects. In [16], artificial intelligence (AI) was used together with information from images and physiological signals to improve the quality of life of older people in nursing homes. The analysis of images and physiological signals enabled AI to propose solutions in different contexts. Authors in [17] introduced SST-EmotionNet, a spatial–spectral–temporal-based network for emotion recognition using EEG; its main advantage is the ability to integrate the features of the spaces mentioned above at the same time. Furthermore, the authors developed a mechanism to discriminate patterns and tested them with two different datasets.

There are few works dedicated to the evaluation of emotional states of people with CP. This topic comprises results from the second set of searches carried out to determine the state of the art. In [18], visually evoked potentials using IAPS imaging were used to analyze evoked emotions in children with CP versus typically developing children. The study was supported by questionnaires in the family environment (KIDSCREEN52) and the Self-Assessment Manikin, administered to the participants who were chosen with the appropriate cognitive level needed to complete this test. The test was carried out with 15 children with CP and 14 children with normal development. It was observed that images with affective content induced lower amplitudes in the brain responses of children with CP compared to those with normal development, which seems to indicate a lower ability to detect emotions. These emotion elicitation techniques will not always be valid, and need to be interpreted with the help of the professionals who attend to the subjects.

In [19], a study with Virtual Reality (VR) was carried out with a 10-year-old girl with a GMFCS (Gross Motor Function Classification System) level of III. The participant’s emotions were analyzed based on facial gestures. In [20], a case study of a girl with cerebral palsy causing communication difficulties with the caregiver was presented. Using a PYTHON image recognition application, the researchers were able to detect patterns that informed the caregiver of their status. In [20], a case study was presented in which the emotional state of a subject with CP was communicated using image recognition. This case attempted to solve the severe communication problems between therapist and patient. The researchers reported limitations related to the lighting required to process the image and the fact that the patient’s disability sometimes made it difficult to detect their emotional state.

Finally, we present previous research in which music is used to induce emotions in subjects. These works show that music can arouse different emotions depending on the musical properties of the piece. In [21], emotions were detected while the subject played a racing video game; the EEG signal was chosen and the classifier was developed by taking the signals from a specific database of emotions. The idea was to insert musical segments into the game according to the emotions detected in the player. In [22], an experiment was carried out where 13 bars with major and minor chords were used as stimuli, different self-assessment scales were used to assess the effect of the musical pieces and the ECG (electrocardiogram) signal and respiration were measured. It was observed that the mode significantly influenced the physiological signals. In [23], an experiment was carried out to see how music affects brain activity, with generated music and classical music as stimuli. Generated music was used so that a possible familiarity did not affect the participant’s emotional state. Using generated music ensures that the emotional state is not based on music that the participant knows from the outset. In this study, classical music was used in search of strong affective responses. EEG and FMRI (Functional Magnetic Resonance Imaging) measurements were taken, and it was observed that the musical stimulus affected the asymmetry in the responses of the two cerebral lobes. In addition to physiological measurements, users also had to respond to tests of their emotional state. In [24], the authors intended to elicit astonishment in users; for this, different musical and Virtual Reality (VR) stimuli were combined. The results were obtained through various self-assessment tests. Physiological measurements were not performed. In addition, as the elicitation of amazement also depends on the personality of the subjects, two additional scales were used; the first measures the predisposition to experience positive emotions and the second measures musical preferences. In [25], an experiment was carried out with 12 people in order to determine the relationship between the EEG signal and emotions. The asymmetry in the value of the EEG signal and its relationship with emotions were studied. To elicit emotions, images from the FACS (Facial Action Coding System) were used to elicit them by imitation and these images were accompanied by music. In [26], the objective was to elicit emotions in the subjects; movie clips were used toward this aim. The clips were chosen to elicit emotional states of happiness, sadness, fear and relaxation. To modify the emotion induced by the scene, music and the color of the ambient light were used for two minutes. In [27], a survey of older people was conducted during the time of the pandemic to assess the role of music as an emotional and social resource. The study shows that as an emotional resource, music induced calm and positive feelings, and as a social resource, it made participants feel accompanied and reminded them of past experiences. In [28], an experiment was designed to determine how music can induce emotions; authors correlated emotions and physiological signals, such as EDA, HR acceleration and others, that showed significant changes between pleasant, neutral and unpleasant 2-second musical extracts.

The reason for conducting this state-of-the-art review in just three lines is due to the absence of studies that have developed measurements for assessing the emotional state of these particular groups. Drawing from existing research in other contexts, various relevant aspects have been evaluated, leading to the formulation of a protocol that amalgamates these findings. This protocol aims to facilitate its application in our specific scenario and with the group of users possessing these unique characteristics. It emerges from years of experience working within these centers, which highlighted the necessity of adapting conventional methods for measuring emotional states and seeking everyday measurement scenarios instead of intentionally inducing emotions, as seen in other contexts described in the literature.

## 3. Materials and Methods

### 3.1. Design of the Study

The first part of the study will be descriptive and observational where the choice of those parameters (dependent variables) that adequately characterize the emotional state of the subjects will be sought. The second part of the study will be analytical, where the influence of music on changes in the selected dependent variables will be determined. In this second part, a single case study will be made, type AB, for each subject, where in condition A the rehabilitation exercise is carried out in silence, while in condition B it is accompanied by motivating music.

### 3.2. Participants

The participants, as already indicated, belong to the ASPACE association (adults) and the Director Mercedes Sanromá Special Education School (children). The study will involve 40 subjects: 20 adults and 20 children. The following criteria are proposed for this group of participants.

#### 3.2.1. Inclusion Criteria

- People with a recognized disability, caused by a disease or permanent health situation.
- Aged between 2 and 65 years.
- Have a degree of functional ability in the mobility domain that is categorized as moderate–low. For adult participants, this will be determined through items related to their motor functionalities according to the International Classification of Functioning, Disability and Health (ICF) [29]. For children, it will be determined using the Gross Motor Function Classification System (GMFCS), ref. [30] and Manual Ability Classification System (MACS) [31]. In [32] a study of this population was conducted and the two scales were homogenized to measure adults and children in the same way.
- People with motivation to use technologies and/or who can use wearable devices during the intervention time.
- People who come weekly to the collaborating centers.

#### 3.2.2. Exclusion Criteria

- Presenting a health situation that is incompatible with the use of technology (e.g., use of respirator, pacemaker, sensitive skin).
- Have a very limited cognitive capacity that prevents the individual from following the instructions for the proper use of assistive technology. For adults, this will be measured through relevant items in the ICF scale, and for children through using the Communication Function Classification System (CFCS) [33].
- Not having adequate human support.
- People with hearing impairments.

#### 3.2.3. Recruitment of Participants

All people who meet the inclusion criteria will be invited to participate in the study. The recruitment of participants will be carried out by the TAIS (Technology for Assistance Integration and Health) research group of the University of Seville, through contact with the collaborating centers. In all cases, a cover letter will be delivered to the managers or directors of the centers with all the information about the project. The participants and/or their representatives will sign the informed consent form.

### 3.3. Instruments of Measurement

#### 3.3.1. Tests and Questionnaires

In this subsection, we present tests and questionnaires to determine the basic state of each participant or those to be used before and after the intervention with the participants. It is important to know the quality of life and emotional situation of the subjects to be measured, with the aim of subsequently contrasting the physiological measurements taken. There are two populations: children and adults. In addition, the cognitive status of the subjects may vary. All of these are factors to be taken into account when applying the measurement instruments. Sometimes, depending on the abilities of individual participants that may prevent them from completing the questionnaires independently, their family members or the professionals of the centers will be responsible for completing the questionnaires.

- ICF for the adult population [29].
- MACS [31] for children.
- GMFCS [30] for children.
- CFCS [33] for children.
- KIDSCREEN Questionnaire (accessed on: https://www.kidscreen.org/english/questionnaires/, accessed on 10 May 2024): Will be used in its 10-item version for the evaluation of the child population; it is an instrument that measures the quality of life related to health.
- Musical Preferences Questionnaire: This measure will ask about songs that motivate the subjects and generate a positive and active emotion. Music serves as a catalyst to enhance the enjoyment of the activity. Hence, understanding the musical preferences of the individual user is crucial. The objective is not to employ a uniform, neutral piece of music for all participants and examine its isolated impact.
- EVEA scale and free text to be filled in by caregivers or relatives. The EVEA scale, according to [26], is consistent and has the ability to detect changes in mood. This scale will be used at the beginning of the data recording once the sensors have been placed and at the end of recording time.

#### 3.3.2. Devices for Recording Physiological Data

**Figure 1:**
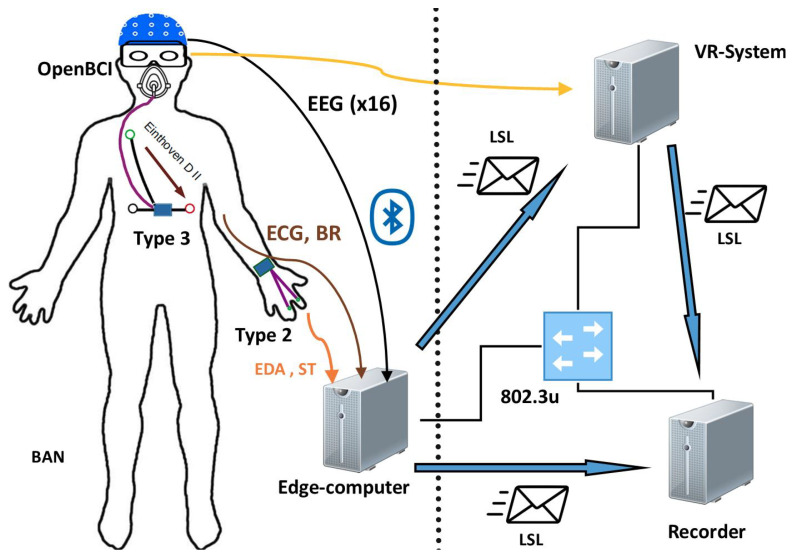
System used in signal recording.

During the sessions, we will use 4 wearable devices developed in the context of this project to collect data. Figure 1 shows a diagram of the sensors that will be used.

These are 4 devices that distribute sensors throughout the body, comprising 4 inertial units on the wrist, ankle, chest and head: a wrist temperature sensor; a sensor of the electrical activity of the skin (EDA) in the phalanges (using dry electrodes placed on the thenar and hypothenar eminences of the dominant hand); an electrocardiography sensor on the chest; and 16 channels of electroencephalography (EEG). In addition, the ambient temperature can be recorded.

**Figure 2:**
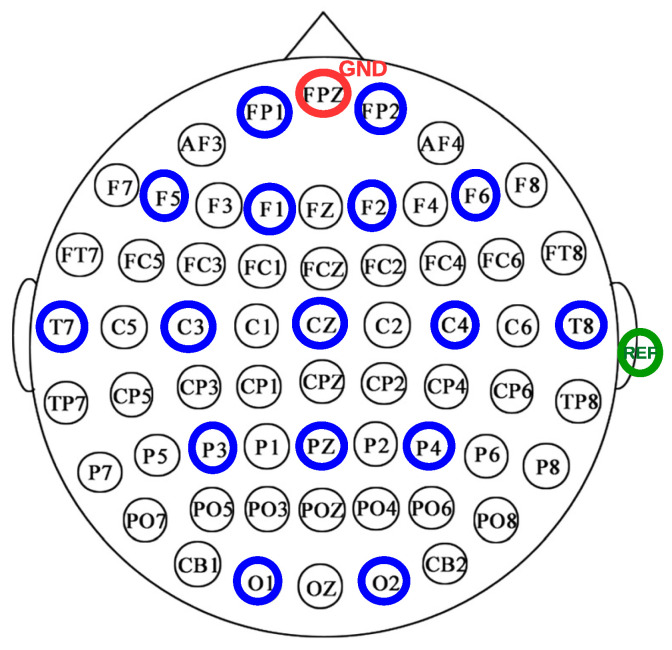
EEG channels selected in the 10/20 system. The position of the ground electrode is marked in red, selected channels in blue, and the reference placed on the earlobe is in green.

One of the wearables we will use is the OpenBCI device (accessed on https://openbci.com/, accessed on 10 May 2024). It is a 16-channel bioamplifier for EEG measurement open-source and low-cost hardware that measures at a maximum rate of 125 Hz when 16 channels are used and includes an inertial unit that measures at 25 Hz. In Figure 2, the electrode position is indicated. The figure shows the positions of the electrodes included in the 10/20 system, which are FP1, FP2, F1, F2, F5, F6, Cz, C3, C4, T7, T8, Pz, P3, P4, O1 and O2. Additional reference and ground electrodes will be placed on the right ear and FPz positions, respectively. The system has been validated in various studies, and the electrode positions it utilizes allow for the measurement of all brain regions, providing sufficient information about the subject’s state.

We will analyze the data using the following independent variables according to the approved clinical trial (accessed on https://clinicaltrials.gov/study/NCT05621057, accessed on 10 May 2024):

- Average kinetic energy measurements (in joules) using inertial sensors to estimate energy expenditure in physical activity.
- Instantaneous heart rate (HR), in seconds. Ag/AgCl electrodes will be used for ECG and processing to extract RR segments from two consecutive beats. The position of the R wave is determined using an appropriate algorithm and then the time difference between two consecutive R waves is calculated. RR segments will be used to generate the heart rate variability (HRV).
- The ratio between the low-frequency and high-frequency (LF/HF) components of HRV. This variable shows the balance between the sympathetic and the parasympathetic nervous systems.
- Standard deviation of NN intervals (*SDNN*), Equation (1), root mean square of successive differences between normal heartbeats (*RMSSD*), Equation (2), and percentage of successive *RR* intervals that differ by more than 50 ms (*pNN50*), Equation (3), as temporal variables of HRV. The term “NN interval” that appears in these measurements is the result of removing outliers from the calculated series of *RR* intervals, which can lead to alterations in the measurements. u(n) is the Heaviside, or unit step function.
  $$\bar{RR}=\frac{1}{N}\sum_{i=1}^{N}RR_{i}$$
  (1) $$SDNN=\sqrt{\frac{1}{N}\sum_{i=1}^{N}{\left(RR_{i}-\bar{RR}\right)}^{2}}$$
  (2) $$RMSSD=\sqrt{\frac{1}{N-1}\sum_{i=2}^{N}{\left(RR_{i}-RR_{i-1}\right)}^{2}}$$
  (3) $$pNN50=\frac{100}{N-1}\sum_{i=2}^{N}u(|RR_{i}-RR_{i-1}\left|-50ms\right)$$
- Skin conductance level (SCL) and skin conductance response (SCR) to detect slow and fast variations in EDA, respectively.
- Fractal dimension (FD) of EEG to compute its complexity using Higuchi’s algorithm.
- The spectrum entropy (SE) of EEG is a tool to determine the EEG complexity. The initial step involves acquiring the power spectral density (PSD). The PSD is then normalized by the number of bins, effectively converting it into a probability density function. Finally, the traditional Shannon entropy for information systems is computed.
- EEG coherence. The interplay among neural systems, functioning within each frequency band, is approximated through EEG coherence. The amplitude of EEG is affected by neural synchronization, while the coherence of signals obtained by a pair of electrodes indicates the uniformity and steadiness of the signal’s amplitude and phase. A time delay in the signal should be observable between two interconnected brain regions, which is interpreted as a phase shift in the frequency domain.

**Figure 3:**
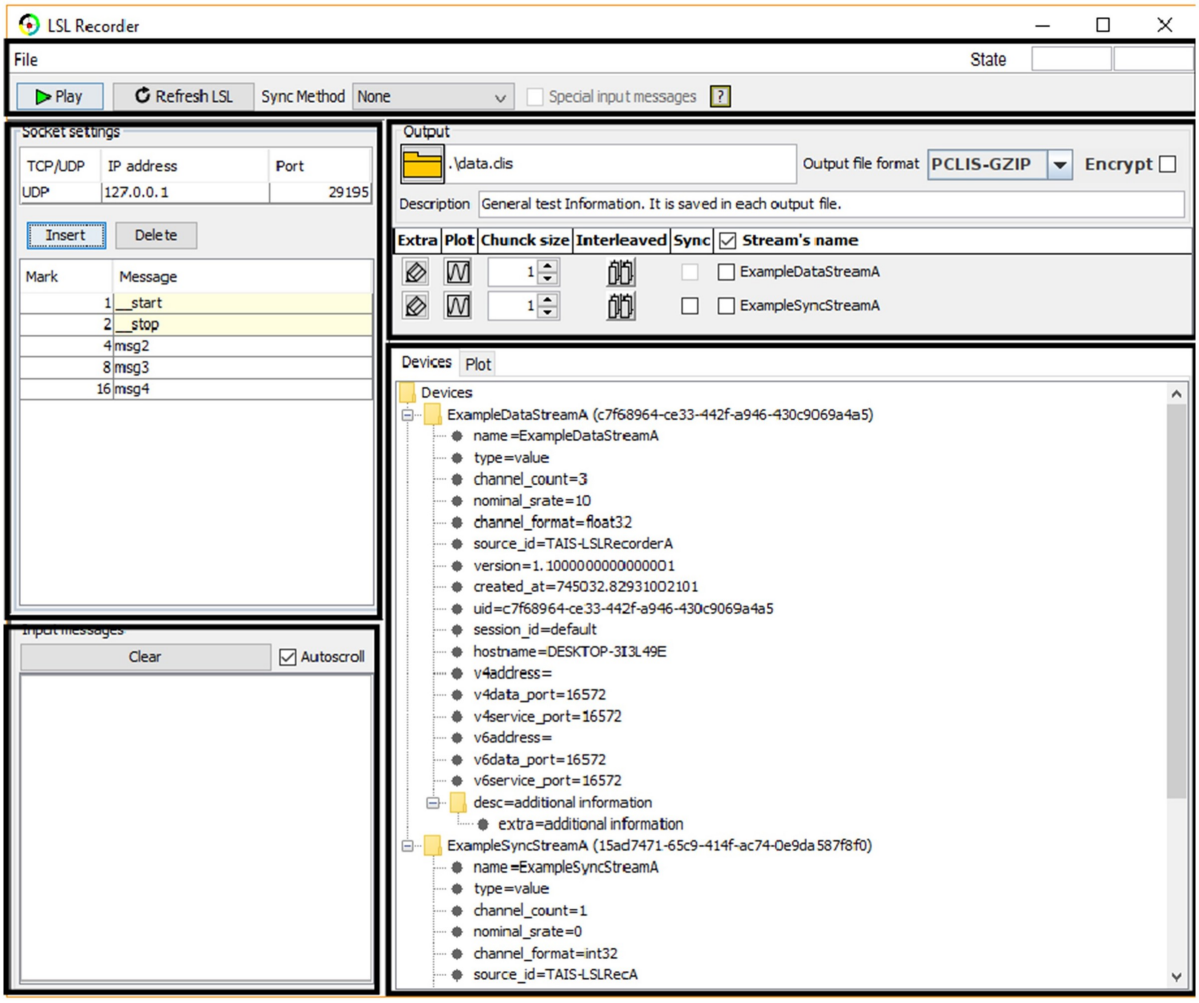
LabRecorder software for synchronized data logging.

Regarding the possibility of muscle activity influencing EEG signal measurements, it is important to note that the muscles engaged in the activity are typically distant from the openBCI, and thus their influence is negligible. EEG is more likely to be affected by facial muscles, head movements, etc. For such cases, Independent Component Analysis (ICA) will be utilized, as it is an efficient algorithm for source separation and artifact removal in EEG signals. The data will be recorded in a synchronized manner using a software application (Figure 3) designed for this purpose [34]. The software utilizes the LabStreaming Layer library. This library supplements each captured data point with a timestamp generated by a high-resolution local clock, simplifying data synchronization. Additionally, the library employs the Network Time Protocol (NTP) to estimate communication delays, thereby adjusting the time offset between clocks.

#### 3.3.3. Contexts and Measurement Frequencies

Four sessions will be held, each divided into two parts:

Selection of dependent variables: The objective of the initial two sessions is to establish a baseline for physiological variables during activities that elicit both positive and negative emotions, to use this baseline as a reference in Part 2. The goal is to minimize the reliance on the EVEA tests, as participants may not always be able to articulate their emotions. The EVEA test serves as a supporting tool for a potential automatic classifier.

Session 1: Measurement of parameters when the subject is conducting a pleasurable daily-life activity in the center.
Session 2: Measurement of parameters when the subject is conducting an uncomfortable daily-life activity in the center.
  These sessions will be determined by conversation with the caregivers since they are specific to each subject.

Half of the participants will start with session 2 and then do session 1, while the rest will follow the reverse order.

Music’s influence on the dependent variables: The aim of the next two sessions is to determine the music’s influence on the dependent variables while conducting rehabilitation exercises.

Session 3: Measurement of subject parameters during the performance of rehabilitation activities in the center.
Session 4: Measurement of subject parameters during rehabilitation activities in the center. This session will be accompanied by music according to the preferences of the subject.

Each participant will select the music to be played in each session according to their musical preference or, failing that, the music will be selected by their caregiver. The rehabilitation activity should be a gentle exercise for the participant, such as pedaling, extending the limbs or any other activity that can be quantified using inertial units. Furthermore, these activities should be determined by the medical/physiotherapist staff according to the capabilities of each volunteer.

**Figure 4:**
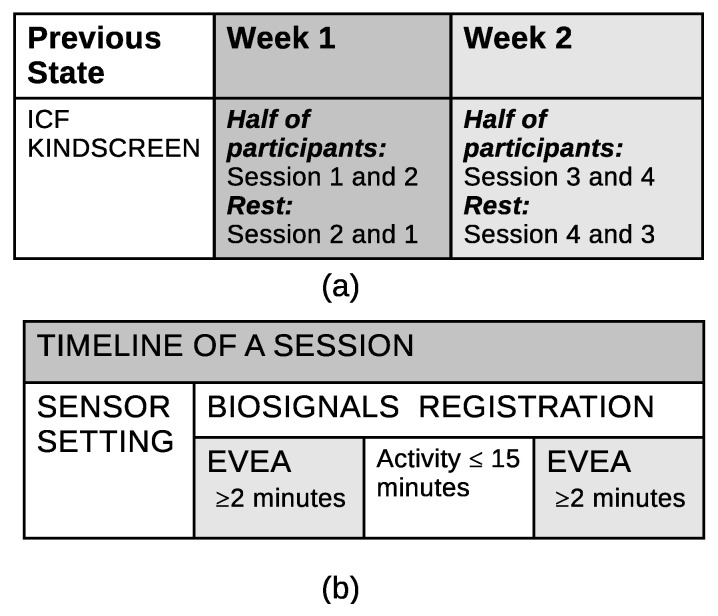
Scheme of the protocol. (**a**) Activities and timing of the global protocol. (**b**) Activities to perform during a session.

Even though every session has a unique theme, the format of the sessions will remain consistent. The sensors will initially be placed on the participant and a data acquisition test will be performed to ensure that all data are being captured correctly. Data recording will initiate while the user is responding to the EVEA test; this phase will be used as a baseline for the session—note that a minimum of two minutes is required. The following phase will be the main activity, with a 15 min time limit, ending with a final EVEA test with identical specifications to the initial one, which will be used as the final baseline. Both baselines allow for the detection of the measurement differentials in each session, alongside the analysis of the participant’s progression during the activity. Each participant is expected to finish the protocol within two weeks, where the first and second sessions will be scheduled for the first week, and the third and fourth sessions will be set for the second one. Figure 4 shows a diagram of the described protocol.

## 4. Statistical Methodology

### 4.1. Sample Size

The population of the centers is approximately 200 individuals (100 from each center, adults and children). According to Cochran’s formula for estimating the sample, assuming, in the worst-case scenario, a value of *p* = *q* = 0.5 (maximum variance), a confidence interval of 95% and a margin of error of 15%, the sample size would be 36 individuals. Therefore, a sample size with 18 adults and 18 children seems adequate.

### 4.2. Data Analysis

In general, numerical variables will be expressed as mean (M) and standard deviation (SD), including range, minimum and maximum.

For the first part of the study, we will compare the dependent variables obtained between sessions 1 and 2 for each subject using the permutation test of the difference in means between different data windows. Those dependent physiological variables that mark significant differences for most of the subjects will be chosen. Changes in EVEA test responses between sessions 1 and 2 will serve as reinforcement for detecting the level of emotional change.

We will also correlate the results of the EVEA test with the base state questionnaires to determine the existence of some type of dependency between the base level of EVEA and its variation with respect to the base state. This dependence will be explored with the Spearman or Pearson correlation test.

For the second part, first, we will analyze the variation in EVEA for sessions 3 and 4 to determine if there is a significant dependence on the independent variable (music) between stages A and B for all the participants. We will use the Kruskal–Wallis test, which does not require any type of assumption regarding the normality and homoscedasticity of the samples. These variations will also be correlated with the quality of life scale (ICF, GMFCS, MACS, CFCS, or KIDSCREEN), as was carried out with the data from the first part.

For the physiological variables and the average kinetic energy, we will determine if their variations are significant between A and B by means of a Kruskal–Wallis test applied to the group of subjects, but also at the individual level by means of a permutation test.

The significance of the tests will be set at three levels, indicated with * (*p* < 0.05), ** (*p* < 0.01) and *** (*p* < 0.001).

## 5. Conclusions

People with CP have particular difficulties in recognizing both their mood and fatigue. This means that in rehabilitation routines or in the performance of physical activity, it is sometimes difficult to properly program the exercise so that they find it motivating and get the most out of the time devoted to such activity.

There are questionnaires that allow the measurement of both the emotional state [35] and the fatigue state [36], but a user with CP does not always have the necessary cognitive and communication skills to rely on these questionnaires.

It is necessary to resort to more objective methods, and the measurement of biosignals is a good alternative. But in order to rely on biosignals, it is necessary to study whether the changes generated in the biosignals are significant. To determine this, it is necessary to put the subjects in contexts where we can predict and control for what their mood is going to be. These contexts are often activities that are part of their daily routines and for which we have experience of their previous responses. The caregivers and the family environment should support the choice of the routines where the biosignals will be recorded. Whenever possible, these measures can be reinforced with tests to measure emotions and physical state by the subjects themselves or their caregivers, in cases where the subject does not have physical or cognitive abilities to complete them. On the other hand, signal processing should be performed to extract parameters that should then be studied statistically to assess their robustness in determining a state. It is also important to assess how physical movement affects a person’s biosignal data. Appropriate techniques should be studied to eliminate the noise produced by this factor so that it does not affect the extraction of accurate information about the condition. To implement the approach outlined in this paper, two sessions, denoted as sessions 1 and 2, have been devised. Session 1 aims to induce a state of pleasant emotion, while session 2 aims to induce discomfort. These sessions enable us to establish correlations between the parameters derived from the inertial sensors or biosignals being recorded and various emotional states. This process helps identify the most reliable parameters to gauge the users’ states across different contexts objectively. Section 3.3.2 enumerates and elucidates all parameters relevant to the various types of recorded signals. Following the proposed data analysis in Section 4.2, we will select the parameters that most effectively discriminate the physical and emotional states of the subjects.

In addition, we want to evaluate how music can be a motivational factor that improves the quality and time dedicated to physical exercise. Therefore, in the proposed experimental protocol, we will introduce an analytical study of type AB in the second part. In Section 2, the review of the state of the art, we presented previous studies with the common finding that musical parameters can induce emotions and the measurement of the EEG signal can be a primary method for showing their change. The proposed protocol will take these factors into account when choosing music and will also introduce the measurement of other types of additional physiological signals.

To operationalize this, sessions 3 and 4 of the experimental protocol have been devised. Music is introduced as a motivational element, and its impact is assessed using the parameters established in sessions 1 and 2. The goal is to enable subjects to invest more time and effort in their rehabilitation sessions, as the achievement of an optimal emotional and physical state during these sessions can be reliably and objectively monitored.

## Figures and Tables

**Table 1:** Questionnaire for users [9]. The choices were yes (Y), no (N), and no answer/do not know (NA).

	Question	Choices
Q1	I liked the activity.	Y, N, NA
Q2	I felt good after the activity.	Y, N, NA
Q3	I felt good before the activity.	Y, N, NA
Q4	I have finished very excited.	Y, N, NA
Q5	I have finished very bored.	Y, N, NA
Q6	I have finished very overwhelmed.	Y, N, NA
Q7	I have finished the activity with pain.	Y, N, NA

**Table 2:** Questionnaire for caregivers [9]. The choices were very low (VL), low (L), normal (N), well (W) and very well (VW).

	Question	Choices
Q1	The care receiver has done the suggested activity as it is described?	VL, L, N, W, VW
Q2	Suggested activity was appropriate for the patient.	VL, L, N, W, VW
Q3	Suggested activity was appropriate at the time it was recommended.	VL, L, N, W, VW

## Data Availability

The data obtained in this research will be hosted in the repository of the University of Seville (https://idus.us.es/).

## Abbreviations

The following abbreviations are used in this manuscript:

AAI	Augmentative Affective Interface
AI	Artificial Intelligence
AIR4DP	Artificial Intelligence and Robotic Assistive Technology devices for Disabled People
AMIGOS	A dataset for Multimodal research of affect, personality traits and mood on Individuals
	and GrOupS
ASPACE	Association of People with Cerebral Palsy of Seville
CFCS	Communication Function Classification System
CP	Cerebral Palsy
ECG	Electrocardiogram
EDA	Electrodermal Activity
EEG	Electroencephalography
EVEA	Scale for Mood Assessment
FACS	Facial Action Coding System
FD	Fractal Dimension
FMRI	Functional Magnetic Resonance Imaging
GMFCS	Gross Motor Function Classification System
GSR	Galvanic Skin Response
HF	High Frequency
HR	Heart Rate
HRV	Heart Rate Variability
ICF	International Classification of Functioning, Disability and Health
LF	Low Frequency
M	Mean
MACS	Manual Ability Classification System
pNN50	Percentage of successive RR intervals that differ by more than 50 ms
PNS	Parasympathetic Nervous System
PSD	Power Spectral Density
RMSSD	Root Mean Square of Successive Differences between normal heartbeats
SCL	Skin Conductance Level
SCR	Skin Conductance Response
SD	Standard Deviation
SDNN	Standard Deviation of NN intervals
SE	Spectral Entropy
SNS	Sympathetic Nervous System
TAIS	Technology for Assistance Integration and Health
VR	Virtual Reality
